# Dose constraints in the rectum and bladder following carbon-ion radiotherapy for uterus carcinoma: a retrospective pooled analysis

**DOI:** 10.1186/s13014-018-1061-7

**Published:** 2018-06-25

**Authors:** Noriyuki Okonogi, Mai Fukahori, Masaru Wakatsuki, Yu Ohkubo, Shingo Kato, Yuhei Miyasaka, Hiroshi Tsuji, Takashi Nakano, Tadashi Kamada

**Affiliations:** 1 National Institute of Radiological Sciences Hospital, National Institutes for Quantum and Radiological Science and Technology, 4-9-1 Anagawa, Inage-ku, Chiba, 263-8555 Japan; 2 Quality Control Section, Clinical Research Cluster, National Institute of Radiological Sciences, National Institutes for Quantum and Radiological Science and Technology, 4-9-1 Anagawa, Inage-ku, Chiba, 263-8555 Japan; 3 Department of Radiology, Jichi Medical University, 3311-1 Yakushiji, Shimotsuke-shi, Tochigi, 329-0498 Japan; 4 Department of Radiation Oncology, Saku Central Hospital Advanced Care Center, 3400-28 Nakagomi, Saku-shi, Nagano, 385-0051 Japan; 5 Department of Radiation Oncology, Saitama Medical University, International Medical Center, 1397-1 yamane. Hidaka-shi, Saitama, 350-1241 Japan; 6 Department of Radiation Oncology, Gunma University Graduate School of Medicine, 3-39-22 Showa-machi, Maebashi, Gunma 371-8511 Japan

**Keywords:** Carbon-ion radiotherapy, Dose constraint, Dose–volume histogram, Late toxicity, Gynecological tumor

## Abstract

**Background:**

Carbon-ion radiotherapy (C-ion RT) provides better dose distribution in cancer treatment compared to photons. Additionally, carbon-ion beams provide a higher biological effectiveness, and thus a higher tumor control probability. However, information regarding the dose constraints for organs at risk in C-ion RT is limited. This study aimed to determine the predictive factors for late morbidities in the rectum and bladder after carbon-ion C-ion RT for uterus carcinomas.

**Methods:**

Between June 1995 and January 2010, 134 patients with uterus carcinomas were treated with C-ion RT with curative intent; prescription doses of 52.8–74.4 Gy (relative biological effectiveness) were delivered in 20–24 fractions. Of these patients, 132 who were followed up for > 6 months were analyzed. We separated the data in two subgroups, a 24 fractions group and a 20 fractions group. Late morbidities, proctitis, and cystitis were assessed according to the Radiation Therapy Oncology Group/European Organisation for Research and Treatment of Cancer criteria. The correlations of clinical and dosimetric parameters, V10–V60, D_5cc_, D_2cc_, and Dmax, with the incidence of ≥grade 1 morbidities were retrospectively analyzed.

**Results:**

In the 24 fractions group, the 3-year actuarial occurrence rates of ≥grade 1 rectal and bladder morbidities were 64 and 9%, respectively. In addition, in the 20 fractions group, the 3-year actuarial occurrence rates of ≥grade 1 rectal and bladder morbidities were 32 and 19%, respectively. Regarding the dose–volume histogram data on the rectum, the D_5cc_ and D_2cc_ were significantly higher in patients with ≥grade 1 proctitis than in those without morbidity. In addition, the D_5cc_ for the bladder was significantly higher in patients with ≥grade 1 cystitis than in those without morbidity. Results of univariate analyses showed that D_2cc_ of the rectum was correlated with the development of ≥grade 1 late proctitis. Moreover, D_5cc_ of the bladder was correlated with the development of ≥grade 1 late cystitis.

**Conclusions:**

The present study identified the dose–volume relationships in C-ion RT regarding the occurrence of late morbidities in the rectum and bladder. Assessment of the factors discussed herein would be beneficial in preventing late morbidities after C-ion RT for pelvic malignancies.

**Trial registration:**

Retrospectively registered (NIRS: 16–040).

**Electronic supplementary material:**

The online version of this article (10.1186/s13014-018-1061-7) contains supplementary material, which is available to authorized users.

## Background

Ion beam therapy, such as protons and carbon ions, provide a superior dose distribution in cancer treatment compared to photons. Additionally, carbon-ion beams are heavier than protons, providing a higher relative biological effectiveness (RBE), and thus a higher tumor control probability, while delivering a smaller dose to the surrounding normal tissues. At our organization, the National Institute of Radiological Sciences (NIRS) in Japan, carbon-ion radiotherapy (C-ion RT) has been used to treat various malignant tumors since 1994.

C-ion RT is a promising treatment for various inoperable and radio-resistant tumors [1–3]. It has been also applied in locally advanced uterus carcinomas. As previous studies show, C-ion RT produces favorable local control without administration of brachytherapy for locally advanced bulky cervical squamous cell carcinoma and cervical adenocarcinoma [4–7]. Considering the anatomical location of the uterus, radiation-induced proctitis or cystitis is the most frequent and critical complication for long-term uterus cancer survivors receiving RT.

In the field of photon radiotherapy, dose–volume histogram (DVH) parameters are predictive factors for late morbidities in many organs [8, 9]. In particular, the Groupe Européen de Curiethérapie and the European Society for Radiotherapy & Oncology has established the dose constraints for organs at risk (OAR) under photon brachytherapy for gynecologic tumors [10, 11]. However, information regarding the dose constraints for the rectum or bladder in C-ion RT remains limited.

In this study, the correlations of clinical and dosimetric parameters with the severity and incidence of late morbidities in the rectum and bladder were retrospectively investigated using data of patients with uterus carcinomas treated with C-ion RT.

## Methods

### Patient characteristics

Between June 1995 and January 2010, 134 patients with uterus carcinomas were treated with C-ion RT alone with curative intent as clinical trials in the NIRS. All patients signed an informed consent form approved by the local institutional review board (NIRS: 16–040). Of these patients, 132 who were followed up for more than 6 months were analyzed in the present study. Among them, 122 had uterine cervical cancer, and 10 had endometrioid adenocarcinoma of the uterus. The median follow-up time was 38 months (range, 6–251 months). The patient and tumor characteristics are shown in Table 1.

**Table 1:** Patient and tumor characteristics

Characteristics (*n* = 132)	
Follow-up period (months)	
Median	38
Range	6–251
Age at diagnosis (years)	
Median age	57
Range	28–85
Diagnosis	
Uterine cervical cancer	122
Uterine endometrial cancer	10
FIGO stage	
II	27
III	86
IVA	19
Histology	
Squamous cell carcinoma	62
Adenosquamous carcinoma	11
Adenocarcinoma	59
Diabetes	
Yes	9
No	123
Anticoagulants	
Yes	11
No	121

Abbreviation: *FIGO* International federation of gynecology and obstetrics

### Carbon-ion radiotherapy

Patients were positioned in customized cradles and immobilized with a low-temperature thermoplastic sheet. A set of 2.5- or 5-mm-thick CT images was taken for three-dimensional treatment planning using HIPLAN software (National Institute of Radiological Sciences, Chiba, Japan). Patients received C-ion RT daily for 4 days/week (Tuesday through to Friday).

The present study is a retrospective analysis consisting of 4 clinical trials [4–7] that were implemented via dose escalation of C-ion RT. The treatment consisted of whole pelvic irradiation and local tumor boost. The clinical target volume (CTV) of whole pelvic irradiation included all areas of gross and potentially microscopic disease, which consisted of the primary site (gross tumor volume, whole uterus, parametrium, and at least the upper half of the vagina and ovaries) and whole pelvic node region (common, internal, and external iliac, obturator, and presacral node regions) (CTV-1). The first planning target volume (PTV-1) included CTV-1 plus a 5-mm safety margin for positioning uncertainty and the uterus plus a 10-mm safety margin for intra- and inter-movement. After completion of whole pelvic irradiation, the CTV included the primary site and swollen lymph nodes (CTV-2). A 5–10-mm margin was added to CTV-2 to produce PTV-2. Finally, the CTV was reduced to include only the gross tumor volume (CTV-3). A 3-mm margin was added to CTV-3 to produce PTV-3. Each PTV was treated by a passive irradiation method with 1–3 fields. If PTV-1 and PTV-2 overlapped with normal tissue structures (e.g., the rectum, sigmoid colon, bladder, and small bowel in the pelvis), priority was given to the PTV coverage. PTV-1 and PTV-2 were covered by ≥90% of the prescribed dose. From 2001, the gastrointestinal (GI) tract was excluded from PTV-3 to limit the dose to the GI tract to a maximum of 60.0 Gy (RBE). The C-ion RT technique has been described in detail elsewhere [4–7]. The radiation dose was calculated for the target volume and surrounding normal structures and was expressed in Gy (relative biological effectiveness [RBE]), which was defined as the physical doses multiplied by the RBE of the C-ions using a semi-empirical and modified microdosimetric kinetic model [12, 13]. The total dose to the tumor ranged 52.8–74.4 Gy (RBE) delivered in 20–24 fractionation schemes. The prescribed C-ion RT doses and fractions are listed in Additional file 1: Table S1 (in the Additional files).

### Follow-up and evaluation of late toxicities

After completing treatment, patients were followed up every 1–3 months for 2 years and every 3 or 6 months thereafter. In the present study, toxicities that occurred 6 months or later after commencing treatment were defined as late toxicities and were graded according to the Radiation Therapy Oncology Group/European Organization for Research and Treatment of Cancer criteria [14]. Late rectal toxicity in patients with suspected rectal or bladder bleeding was confirmed via endoscopic examinations.

### DVH analysis and data collection

DVH analysis was performed to identify predictive factors of late toxicities. For all cases, contours of the outer bladder wall and rectum were generated. The contour of the rectum began at the anorectal junction and ended at the rectosigmoid flexure. The original treatment data were calculated using the treatment planning system HIPLAN. The dose–distribution calculation algorithm of HIPLAN adopted the parallel broad beam method, which could not reproduce blurring and non-uniformity of distributions in the irradiation fields. A high-precision treatment planning system, that is, XiO-N, was developed jointly by NIRS, Mitsubishi Electric Corporation, and Elekta. All DVH data were recalculated using XiO-N in the present study. Based on DVH data obtained from the XiO-N software, the following parameters regarding the rectum or bladder were extracted for each patient: V10, V20, V30, V40, V50, V60, the maximum dose (Dmax), the minimal radiation doses for the most irradiated volumes of 2 cm^3^ (D_2cc_), and D_5cc_. Following this, we assessed the relationships between these parameters and late toxicities. Although C-ion RT is less sensitive to fractionation than photon RT, we separated the data in two subgroups, a 24 fractions group and a 20 fractions group.

### Statistical analysis

The follow-up time was calculated from the first date of irradiation. The Mann–Whitney U test was used to compare the irradiated volumes of OARs. Cumulative incidents of late toxicities were evaluated using the Kaplan–Meier method and compared via the log-rank test as a univariate analysis among the different subgroups. Furthermore, some factors with significance (*p* < 0.05) in the univariate analysis were applied to the multivariate analysis using the Cox proportional hazard model. Differences were considered significant if the *p* value was below 0.05. All statistical analyses were performed using SPSS 22.0 for Mac (SPSS, Chicago, IL, USA).

## Results

### Incidence of late toxicities in the rectum and bladder

The incidences of late toxicities in the rectum and bladder in all the 132 patients are summarized in Table 2. Representative endoscopic image scored as grade 2 rectal toxicity and the dose distribution are shown in Additional file 2: Figure S1. The median interval between the beginning of treatment and the development of any rectal morbidity was 12 months (range, 6–67 months) and that of bladder morbidities was 19 months (range, 6–98 months).

**Table 2:** Late maximal morbidities in rectum and bladder by RTOG/EORTC scoring in 132 patients

RTOG/EORTC Grade					
24 fractions (*n* = 41)	0	1	2	3	4
Rectum	18	12	3	1	7
Bladder	36	2	2	0	1
20 fractions (*n* = 91)	0	1	2	3	4
Rectum	66 (53)	20 (12)	4 (3)	0 (0)	1 (1)
Bladder	74 (57)	8 (6)	9 (6)	0 (0)	0 (0)

Abbreviation:

*RTOG/EORTC* Radiation therapy oncology group/European organization for research and treatment of cancer

The number in brackets indicates the number of patients who were treated with a previous dose constraint of <60 Gy (RBE), maximal dose, to the GI tract

**Fig. 1:**
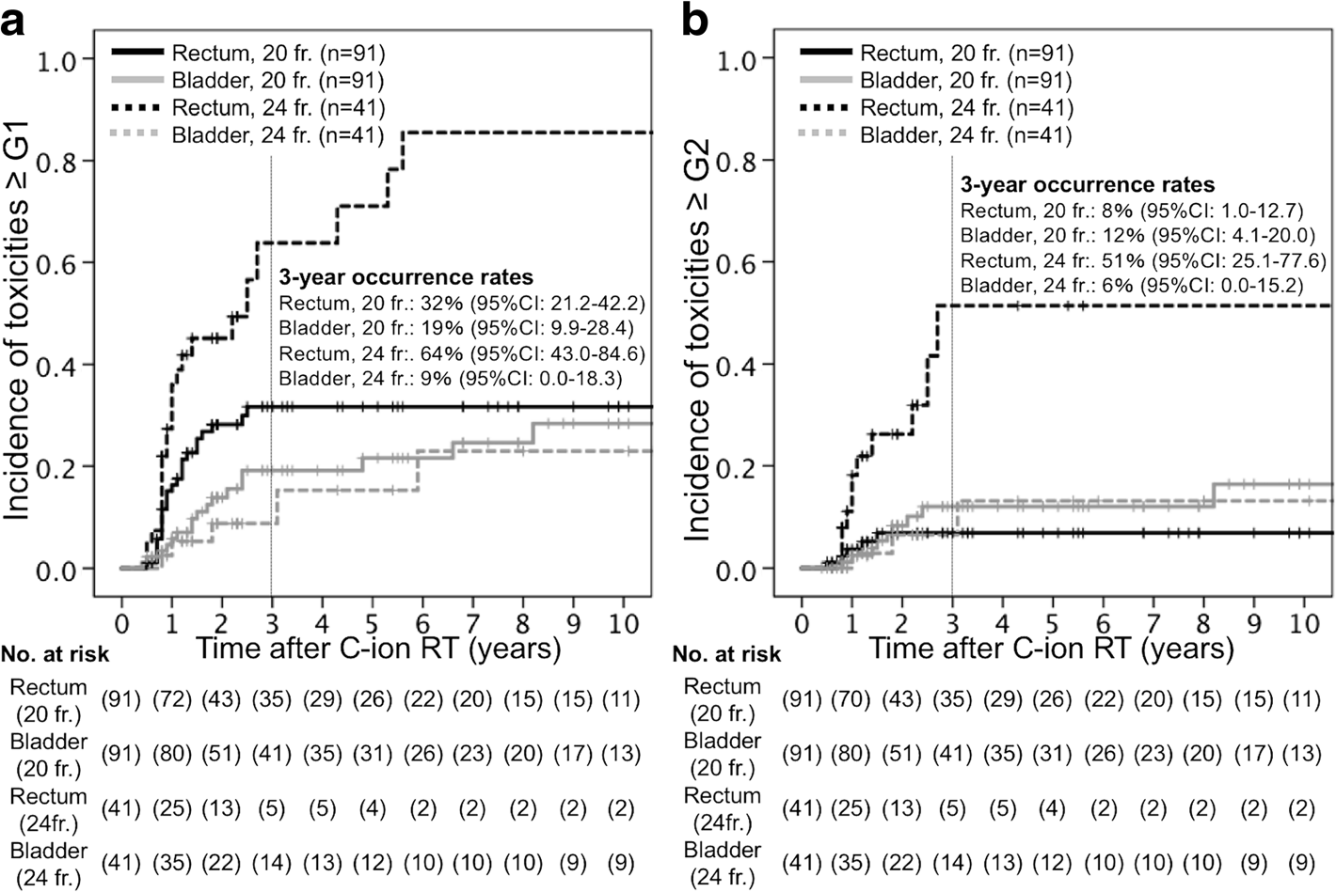
Cumulative incidence curves of late morbidities in the rectum and bladder. The incidence curves of ≥grade 1 toxicities in the rectum and bladder are shown in Fig. 1a, and the incidence curves of ≥grade 2 toxicities in the rectum and bladder are shown in Fig. 1b. Solid lines show the data for the 20 fractions group and dotted lines show the data for the 24 fractions group. *Abbreviation: CI = confidence interval*

In the 24 fractions group, the 3-year actuarial occurrence rates of ≥grade 1 rectal and bladder morbidities were 64 and 9%, respectively. In addition, the 3-year actuarial occurrence rates of ≥grade 2 rectal and bladder morbidities were 54 and 6%, respectively. In the 20 fractions group, the 3-year actuarial occurrence rates of ≥grade 1 rectal and bladder morbidities were 32 and 19%, respectively. In addition, the 3-year actuarial occurrence rates of ≥grade 2 rectal and bladder morbidities were 8 and 12%, respectively (Fig. 1). All DVH data for 132 cases are shown in Additional file 3: Figure S2 and Additional file 4: Figure S3. The main symptom of rectal toxicity was bleeding. Three patients had grade 3 rectal morbidity and underwent elective surgery, while 8 patients had grade 4 morbidity and underwent emergency surgery. Meanwhile, the main symptom of bladder toxicity was hematuria, not urgency or incontinence. One patient with grade 4 cystitis had vesicovaginal fistula. All the patients with severe toxicities were surgically treated. All grade 2 or higher morbidities were observed within 36 months after C-ion RT, except for 2 cases of cystitis. The details of patients developed grade 2 or higher morbidities are shown in Additional file 5: Table S2.

### Correlation of DVH parameters and late toxicities

DVHs classified by the presence or absence of morbidities are shown in Additional file 6: Figure S4 and Additional file 7: Figure S5. Table 3 summarizes the DVH parameters from V10 to V60, D_5cc_, D_2cc_, and Dmax between the patients with grade 1 or higher morbidity and those without morbidity. There were no significant differences between dosimetric parameters with the late morbidities in the rectum and bladder in the 24 fractions group. Regarding the DVH data in the 20 fractions group, the D_5cc_ and the D_2cc_ for the rectum were significantly higher in patients with ≥grade 1 proctitis than in those without toxicity. In addition, the D_5cc_ for the bladder was significantly higher in patients with ≥grade 1 cystitis than in those without toxicity. However, a correlation between dosimetric parameters and the severity of morbidities was not found in the 20 fractions group. As shown in Table 2 and Additional file 5: Table S2, seven patients in the 24 fractions group developed Grade 4 toxicity in the rectum. There was no statistical significance in any DVH parameter, including D_2cc_, between the patients with grade 4 proctitis (*n* = 7) and those with ≤grade 3 proctitis (Additional file 8: Table S3). However, all the parameters were higher in patients with grade 4 proctitis compared to those with ≤grade 3 proctitis.

**Table 3:** Correlation between dose-volume histogram parameters and ≥ grade 1 late morbidities in rectum and bladder

	24 fractions			20 fractions		
	Grade 0	≥Grade 1	*p* value	Grade 0	≥Grade 1	*p* value
Rectum	*n* = 18	*n* = 23		*n* = 66	*n* = 25	
V10 (mean ± SD, cc)	36.0 ± 22.9	46.7 ± 26.0	0.118	32.8 ± 11.6	26.3 ± 10.5	0.096
V20 (mean ± SD, cc)	34.2 ± 22.5	45.8 ± 25.8	0.153	28.1 ± 10.6	26.0 ± 10.1	0.397
V30 (mean ± SD, cc)	29.1 ± 21.1	34.9 ± 18.4	0.365	19.8 ± 9.4	20.4 ± 9.3	0.807
V40 (mean ± SD, cc)	20.5 ± 16.0	24.7 ± 15.9	0.421	12.6 ± 8.5	14.7 ± 8.6	0.294
V50 (mean ± SD, cc)	11.3 ± 11.0	15.5 ± 11.7	0.255	6.3 ± 6.2	8.4 ± 5.8	0.156
V60 (mean ± SD, cc)	5.1 ± 9.1	7.7 ± 7.7	0.332	1.2 ± 2.1	1.8 ± 2.0	0.262
D5 cc [mean ± SD, Gy (RBE)]	54.7 ± 7.4	57.1 ± 8.5	0.349	48.1 ± 8.3	52.1 ± 6.2	0.017
D2 cc [mean ± SD, Gy (RBE)]	59.1 ± 7.0	61.7 ± 6.8	0.242	53.7 ± 6.8	57.3 ± 5.3	0.011
Dmax [mean ± SD, Gy (RBE)]	65.0 ± 7.2	67.0 ± 6.4	0.360	66.7 ± 4.7	68.3 ± 4.6	0.160
Bladder	*n* = 36	*n* = 5		*n* = 74	*n* = 17	
V10 (mean ± SD, cc)	104.4 ± 78.4	108.0 ± 94.0	0.928	150.3 ± 67.6	135.3 ± 63.9	0.410
V20 (mean ± SD, cc)	98.3 ± 74.8	91.8 ± 61.8	0.858	124.1 ± 59.7	121.6 ± 59.4	0.876
V30 (mean ± SD, cc)	71.0 ± 45.9	67.9 ± 43.4	0.890	98.3 ± 52.4	101.9 ± 57.0	0.808
V40 (mean ± SD, cc)	55.9 ± 37.3	53.5 ± 38.8	0.897	64.2 ± 36.2	76.0 ± 48.8	0.265
V50 (mean ± SD, cc)	38.3 ± 27.9	38.5 ± 30.3	0.988	34.9 ± 24.2	48.0 ± 30.4	0.061
V60 (mean ± SD, cc)	25.5 ± 22.5	20.4 ± 15.0	0.638	13.3 ± 11.7	18.8 ± 13.7	0.097
D5 cc [mean ± SD, Gy (RBE)]	62.9 ± 7.8	65.0 ± 7.4	0.577	61.9 ± 7.3	64.8 ± 6.4	0.041
D2 cc [mean ± SD, Gy (RBE)]	65.3 ± 6.9	66.2 ± 7.8	0.819	64.9 ± 6.4	66.9 ± 5.8	0.117
Dmax [mean ± SD, Gy (RBE)]	67.8 ± 7.8	69.4 ± 8.2	0.688	70.1 ± 4.8	70.4 ± 4.2	0.774

Univariate analyses were then performed for clinical and DVH parameters to analyze their effect on the development of late morbidities in the rectum and bladder. Patients were divided into 2 groups according to median values for the analysis of both age and DVH parameters. On univariate analysis for rectum morbidity, V60 and D_2cc_ were found to correlate with the development of proctitis, and D_2cc_ was the most significant predictive factor for proctitis in the 20 fractions group. In addition, none of the dosimetric parameters correlated with the development of proctitis in the 24 fractions group (Table 4). Regarding the univariate analysis for bladder morbidity, D_5cc_ and diabetes were found to correlate with the development of cystitis in the 20 fractions group (Table 5). The multivariate analysis showed that D_5cc_ was an independent risk factor for occurrence of cystitis in this group (*p* = 0.030, hazard ratio = 3.497, 95% confidence interval = 1.126–10.867). In addition, none of the parameters correlated with the development of cystitis in the 24 fractions group.

**Table 4:** Univariate analysis for risk factors of ≥grade 1 late morbidities in rectum

	24 fractions, *n* = 41			20 fractions, *n* = 91		
Factors	Subgroup	Number of patients	*p* value	Subgroup	Number of patients	*p* value
V10	≥37.1 cm^3^	21	0.755	≥30.0 cm^3^	46	0.057
	< 37.1 cm^3^	20		< 30.0 cm^3^	45	
V20	≥36.9 cm^3^	21	0.960	≥26.2 cm^3^	44	0.185
	< 36.9 cm^3^	20		< 26.2 cm^3^	47	
V30	≥25.5 cm^3^	21	0.615	≥18.3 cm^3^	46	0.921
	< 25.5 cm^3^	20		< 18.3 cm^3^	45	
V40	≥16.9 cm^3^	21	0.668	≥11.2 cm^3^	45	0.354
	< 16.9 cm^3^	20		< 11.2 cm^3^	46	
V50	≥8.4 cm^3^	21	0.437	≥5.4 cm^3^	45	0.050
	< 8.4 cm^3^	20		< 5.4 cm^3^	46	
V60	≥3.0 cm^3^	15	0.337	≥0.5 cm^3^	43	0.038
	< 3.0 cm^3^	15		< 0.5 cm^3^	48	
D_5cc_	≥55.1 Gy (RBE)	21	0.429	≥50.6 Gy (RBE)	46	0.052
	< 55.1 Gy (RBE)	20		< 50.6 Gy (RBE)	45	
D_2cc_	≥59.6 Gy (RBE)	21	0.254	≥56.1 Gy (RBE)	46	0.027
	< 59.6 Gy (RBE)	20		< 56.1 Gy (RBE)	45	
Dmax	≥68.0 Gy (RBE)	21	0.086	≥67.0 Gy (RBE)	46	0.517
	< 68.0 Gy (RBE)	20		< 67.0 Gy (RBE)	45	
Diabetes	Yes	2	0.823	Yes	7	0.353
	No	39		No	84	
Anticoagulants	Yes	1	0.196	Yes	10	0.458
	No	40		No	81	
Age	≥56 years	21	0.727	≥60 years	47	0.464
	< 56 years	20		< 60 years	44

**Table 5:** Univariate analysis for risk factors of ≥grade 1 late morbidities in bladder

	24 fractions, *n* = 41			20 fractions, *n* = 91		
Factors	Subgroup	Number of patients	*p* value	Subgroup	Number of patients	*p* value
V10	≥79.8 cm^3^	21	0.698	≥154.5 cm^3^	46	0.194
	< 79.8 cm^3^	20		< 154.5 cm^3^	45	
V20	≥76.7 cm^3^	20	0.687	≥121.1 cm^3^	45	0.562
	< 76.7 cm^3^	21		< 121.1 cm^3^	46	
V30	≥56.5 cm^3^	21	0.790	≥93.9 cm^3^	46	0.790
	< 56.5 cm^3^	20		< 93.9 cm^3^	45	
V40	≥48.5 cm^3^	21	0.757	≥61.2 cm^3^	45	0.281
	< 48.5 cm^3^	20		< 61.2 cm^3^	46	
V50	≥32.5 cm^3^	21	0.574	≥32.0 cm^3^	46	0.243
	< 32.5 cm^3^	20		< 32.0 cm^3^	45	
V60	≥20.1 cm^3^	15	0.845	≥11.5 cm^3^	44	0.054
	< 20.1 cm^3^	15		< 11.5 cm^3^	44	
D_5cc_	≥63.7 Gy (RBE)	21	0.487	≥63.8 Gy (RBE)	46	0.011
	< 63.7 Gy (RBE)	20		< 63.8 Gy (RBE)	45	
D_2cc_	≥66.8 Gy (RBE)	21	0.487	≥65.8 Gy (RBE)	46	0.288
	< 66.8 Gy (RBE)	20		< 65.8 Gy (RBE)	45	
Dmax	≥71.0 Gy (RBE)	21	0.057	≥71.0 Gy (RBE)	48	0.631
	< 71.0 Gy (RBE)	20		< 71.0 Gy (RBE)	43	
Diabetes	Yes	2	0.543	Yes	7	0.003
	No	39		No	84	
Anticoagulants	Yes	1	0.603	Yes	10	0.812
	No	40		No	81	
Age	≥56 years	21	0.489	≥60 years	47	0.519
	< 56 years	20		< 60 years	44	

## Discussion

C-ion RT has shown promising treatment results in patients with locally advanced uterus carcinomas [4–7]. However, although late morbidities are the most critical problems for long-term survivors, few reports demonstrate a relationship between late morbidities in the rectum or bladder and DVH parameters in C-ion RT. In patients treated with C-ion RT in a previous clinical trial, those in whom the dose exceeded 60 Gy (RBE) in the intestines developed major toxicities [5]. Thus, we reported that in C-ion RT for uterus carcinoma, the maximal dose to the intestines, including the rectum and sigmoid colon, should be limited to < 60 Gy (RBE) to avoid major toxicities [5]. Although patients with high-grade toxicity tended to have received more radiation dose to the rectum, no significant differences among the DVHs were noted in the previous study. In the present study, there were no significant differences between dosimetric parameters with the late morbidities in the rectum and bladder in the 24 fractions group. However, we identified the dose–volume relationships with regard to the occurrence of late morbidities in the rectum and bladder in uterus carcinomas in the 20 fractions group.

The incidence of ≥grade 2 rectal morbidity was relatively higher in the 24 fractions group in the present study. Among the four clinical trials, the earlier two trials were conducted with 24 fractions of C-ion RT and the latter two were conducted with 20 fractions of C-ion RT. Overall, there was no difference in preparation of the patients or rectal filling between the two groups. However, there was no information regarding dose constraint for OARs when the patients were treated in 24 fractions. This may be the main reason for the higher incidence of ≥grade 2 rectal morbidity in the 24 fractions group.

Regarding the predictors of morbidities, univariate analysis suggested that V60 and D_2cc_ were significantly associated with the development of late rectal toxicities. Moreover, we found that D_2cc_ was the most significant predictive factor for late rectal morbidities, the risk of which can be reduced by maintaining the D_2cc_ below 57.3 Gy (RBE). Meanwhile, univariate analysis suggested that the risk of late bladder morbidities can be reduced by maintaining the D_5cc_ below 64.8 Gy (RBE). The DVHs classified by severity of cystitis (Additional file 7: Figure S5) showed that the radiation dose less than 20 Gy (RBE) in patients without toxicities was higher than in those with ≥grade 1 cystitis. This result indicates that a dose less than 20 Gy (RBE) to the bladder may not induce cystitis.

Previously, Ishikawa et al. reported that V50 of the rectum was an independent risk factor for the occurrence of late rectal morbidity toxicity after C-ion RT for prostate cancer [15]. Meanwhile, we found that D_2cc_ was the most significant predictive factor for late rectal morbidities. The reason for the discrepancy in the results between the study by Ishikawa et al. and the present study may be the accuracy of the planning software. Ishikawa et al. evaluated the DVH data obtained from the HIPLAN software. The dose–distribution calculation algorithm of HIPLAN could not reproduce blurring and non-uniformity of distributions in the irradiation fields. Considering the dose calculating limitation of the HIPLAN software, we recalculated all DVH data using XiO-N in the present study. Furthermore, Fukahori et al. recently reported that rectal complication in prostate cancer after carbon-ion RT would more likely be caused by high dose irradiation to a small volume of the rectum [16]. Taken together with these results, the dose constraint in small volumes such as D_2cc_ is a reliable predictive factor for late rectal morbidities in C-ion RT.

Regarding the dose constraints in photon beam therapy, growing evidence supports that D_2cc_ is one of the most reliable index that predict late morbidities in the rectum or bladder [17–21]. Mazeron et al. reported that the equieffective D_2cc_ for a 10% probability for overall rectal ≥grade 2 toxicity was 69.5 Gy [20]. In the analysis, they concluded that a D_2cc_ ≤ 65 Gy is associated with more minor and less frequent rectal morbidity, whereas a D_2cc_ ≥ 75 Gy is associated with more major and more frequent rectal morbidity. As for bladder morbidity, Kim et al. reported that grade ≥ 2 bladder morbidities occurred in significantly fewer patients with a D_2cc_ of ≤95 Gy than in patients with a D_2cc_ of > 95 Gy [21]. Further data on dose–volume relationships in cervical cancer radiotherapy are expected from an ongoing prospective multicenter study in cervical cancer [22].

The values of D_2cc_ for rectal morbidity, or the index for bladder morbidity differ between the present study and the studies previously reported in photon therapy. These discrepancies can be attributed to the difference in treatment periods, but differences in linear energy transfer (LET) should also be considered. In charged-particle therapy treatment planning, a clinically relevant dose, i.e., a biological dose, which is defined as the product of absorbed dose and the RBE, is optimized for the individual clinical case. The RBE is a dose ratio of a reference radiation to that of the radiation of interest that yields a defined biological response, and it depends on various factors, such as LET, endpoint, and tissue type [23]. This is however, difficult due to the complexity of the biological effect mechanism. In Japan, the survival of human salivary gland tumor cells has been used as the endpoint for the RBE of C-ion beams, and it is used for all types of tumors and normal tissues [12, 13]. The generic RBE of 3.0 is thus clinically used today in C-ion RT, irrespective of these factors [12]. However, the LET value in the target volume in C-ion RT is heterogeneous in the clinical setting [24]. Considering the complexities described above, the dose constraints of photon therapy cannot be adopted reliably as done for C-ion RT. Hence, it might be better to evaluate dose constraints from clinical trials, such as this study.

The findings and the analyses in the present study are based on a cohort from 4 major clinical trials of C-ion RT for locally advanced uterus carcinomas. As such, we identified reliable indices that predict late morbidities in the rectum or bladder. However, our study has some limitations other than its retrospective nature. First, the toxicities consisted mainly of bleeding or fistula, and not stenosis nor atrophy. Therefore, the relationship between intermediate and large volume dose parameters and late morbidities may not have been identified. Second, uncertainties exist in assessing the delivered dose in our study. The prescribed doses were used to assess the dose–effect relationships, assuming that the prescribed doses are equivalent to delivered doses. Third, the data we showed were based on the fixed RBE model calculation. The dose constraints in the present study cannot be applied in carbon facilities using a local effect model [25]. Hence, we are now conducting concurrent chemotherapy with C-ion RT, and these factors can significantly impact this correlation.

## Conclusions

The present study identified the dose–volume relationships in C-ion RT regarding the occurrence of late morbidities in the rectum and bladder. In recent years, the number of C-ion RT facilities has increased, and almost all institutions apply a similar schedule of 4 days of irradiation per week. Although several limitations remain, our data may be beneficial for radiation oncologists who are treating patients with C-ion RT for other pelvic malignancies. Further multivariate analyses are required for a more comprehensive understanding of the correlation between late morbidities and radiation dose and for developing nomograms by taking into account all pertinent parameters, allowing for individualization of dose prescription.

## Additional files

Additional file 1: **Table S1.** Dose fractions of C-ion RT and the number of patients with morbidities for each prescription dose. The number in brackets indicates the number of patients who were treated with a previous dose constraint of < 60 Gy (RBE), maximal dose, to the GI tract. For those patients with both rectum and bladder morbidities, the patient was categorized as having a higher grade cancer in this table. (DOCX 32 kb)

Additional file 2: **Figure S1.** Representative endoscopic image of proctitis and isodose curves of carbon-ion radiotherapy of the patient. (A) Representative endoscopic image of grade 2 proctitis and (B) isodose curves of carbon-ion radiotherapy on a sagittal computed tomography image of the patient. This patient developed Grade 2 proctitis on the anterior rectal wall 1 year after carbon-ion radiotherapy. Yellow arrows indicate the proctitis region. Highlighted are 95% (red), 90% (orange), 80% (light purple), 70% (pink), 50% (green), 30% (green), and 10% (purple) isodose curves. (TIFF 9568 kb)

Additional file 3: **Figure S2.** Dose–volume histograms of the rectum. (A) Dose–volume histograms (DVHs) of the rectum in 41 patients who received carbon-ion radiotherapy in 24 fractions and (B) DVHs of the rectum in 91 patients who received carbon-ion radiotherapy in 20 fractions. Each line shows the data for each patient and each color indicates the severity of proctitis; Grade 0 (cyan), Grade 1 (green), Grade 2 (yellow), Grade 3 (red), and Grade 4 (brown). *Abbreviation: Gr = Grade, RBE = relative biological effectiveness. (TIFF 9568 kb)*

Additional file 4: **Figure S3.** Dose–volume histograms of the bladder. (A) Dose–volume histograms (DVHs) of the bladder in 41 patients who received carbon-ion radiotherapy in 24 fractions and (B) DVHs of the bladder in 91 patients who received carbon-ion radiotherapy in 20 fractions. Each line shows the data for each patient and each color indicates the severity of cystitis; Grade 0 (cyan), Grade 1 (green), Grade 2 (yellow), Grade 3 (red), and Grade 4 (brown). *Abbreviation: Gr = Grade, RBE = relative biological effectiveness*. (TIFF 9568 kb)

Additional file 5: **Table S2.** List of patients who developed grade 2 or higher morbidities. Abbreviation: UCC: Uterine cervical carcinoma, UEA: Uterine endometrioid adenocarcinoma. The asterisks indicates the number of patients who were treated with a previous dose constraint of < 60 Gy (RBE), maximal dose, to the GI tract. (DOCX 19 kb)

Additional file 6: **Figure S4.** Comparisons of dose–volume histograms of the rectum. Comparisons of dose–volume histograms of the rectum (A) in 41 patients who received carbon-ion radiotherapy in 24 fractions and (B) in 91 patients who received carbon-ion radiotherapy in 20 fractions. Each line shows the average value and each color indicates the presence or absence of proctitis; Grade 0 (cyan) and ≥ Grade 1 (red). Solid lines show the averages and dotted lines show the ±1 SD. *Abbreviation: SD = standard deviation*. (TIFF 9568 kb)

Additional file 7: **Figure S5.** Comparisons of dose–volume histograms of the bladder. Comparisons of dose–volume histograms of the bladder (A) in 41 patients who received carbon-ion radiotherapy in 24 fractions and (B) in 91 patients who received carbon-ion radiotherapy in 20 fractions. Each line shows the average value and each color indicates the presence or absence of cystitis; Grade 0 (cyan) and ≥ Grade 1 (red). Solid lines show the averages and dotted lines show the ±1 SD. *Abbreviation: SD = standard deviation. (TIFF 9568 kb)*

Additional file 8: **Table S3.** Correlation between dose-volume histogram parameters and late morbidities of the rectum in the 24 fractions group. This table summarizes the dose–volume histogram parameters from V10 to V60, D_5cc_, D_2cc_, and Dmax according to the grading of rectal morbidity in the 24 fractions group. (DOCX 16 kb)

## Abbreviations

C-ion RT: Carbon-ion radiotherapy
DVH: Dose–volume histogram
LET: Linear energy transfer
NIRS: National Institute of Radiological Sciences
RBE: Relative biological effectiveness

## References

[1] Tsujii H, Kamada T (2012). A review of update clinical results of carbon ion radiotherapy. Jpn J Clin Oncol.

[2] Mizoe JE, Hasegawa A, Jingu K, Takagi R, Bessyo H, Morikawa T (2012). Results of carbon ion radiotherapy for head and neck cancer. Radiother Oncol.

[3] Sugahara S, Kamada T, Imai R, Tsuji H, Kameda N, Okada T (2012). Carbon ion radiotherapy for localized primary sarcoma of the extremities: results of a phase I/II trial. Radiother Oncol.

[4] Nakano T, Suzuki M, Abe A, Suzuki Y, Morita S, Mizoe J (1999). The phase I/II clinical study of carbon ion therapy for cancer of the uterine cervix. Cancer J Sci Am.

[5] Kato S, Ohno T, Tsujii H, Nakano T, Mizoe JE, Kamada T (2006). Dose escalation study of carbon ion radiotherapy for locally advanced carcinoma of the uterine cervix. Int J Radiat Oncol Biol Phys.

[6] Wakatsuki M, Kato S, Ohno T, Karasawa K, Ando K, Kiyohara H (2014). Dose-escalation study of carbon ion radiotherapy for locally advanced squamous cell carcinoma of the uterine cervix (9902). Gynecol Oncol.

[7] Wakatsuki M, Kato S, Ohno T, Karasawa K, Kiyohara H, Tamaki T (2014). Clinical outcomes of carbon ion radiotherapy for locally advanced adenocarcinoma of the uterine cervix in phase 1/2 clinical trial (protocol 9704). Cancer.

[8] Emami B, Lyman J, Brown A, Coia L, Goitein M, Munzenrider JE (1991). Tolerance of normal tissue to therapeutic irradiation. Int J Radiat Oncol Biol Phys.

[9] Marks LB, Yorke ED, Jackson A, Ten Haken RK, Constine LS, Eisbruch A (2010). Use of normal tissue complication probability models in the clinic. Int J Radiat Oncol Biol Phys.

[10] Haie-Meder C, Pötter R, Van Limbergen E, Briot E, De Brabandere M, Dimopoulos J (2005). Recommendations from Gynaecological (GYN) GEC-ESTRO working group (I): concepts and terms in 3D image based 3D treatment planning in cervix cancer brachytherapy with emphasis on MRI assessment of GTV and CTV. Radiother Oncol.

[11] Pötter R, Haie-Meder C, Van Limbergen E, Barillot I, De Brabandere M, Dimopoulos J (2006). Recommendations from gynaecological (GYN) GEC ESTRO working group (II): concepts and terms in 3D image-based treatment planning in cervix cancer brachytherapy—3D dose volume parameters and aspects of 3D image-based anatomy, radiation physics, radiobiology. Radiother Oncol.

[12] Kanai T, Endo M, Minohara S, Miyahara N, Koyama-ito H, Tomura H (1999). Biophysical characteristics of HIMAC clinical irradiation system for heavy-ion radiation therapy. Int J Radiat Oncol Biol Phys.

[13] Inaniwa T, Kanematsu N, Matsufuji N, Kanai T, Shirai T, Noda K (2015). Reformulation of a clinical-dose system for carbon-ion radiotherapy treatment planning at the National Institute of Radiological Sciences, Japan. Phys Med Biol.

[14] Cox JD, Stetz J, Pajak TF (1995). Toxicity criteria of the radiation therapy oncology group (RTOG) and the European Organization for Research and Treatment of Cancer (EORTC). Int J Radiat Oncol Biol Phys.

[15] Ishikawa H, Tsuji H, Kamada T, Hirasawa N, Yanagi T, Mizoe JE (2006). Risk factors of late rectal bleeding after carbon ion therapy for prostate cancer. Int J Radiat Oncol Biol Phys.

[16] Fukahori M, Matsufuji N, Himukai T, Kanematsu N, Mizuno H, Fukumura A (2016). Estimation of late rectal normal tissue complication probability parameters in carbon ion therapy for prostate cancer. Radiother Oncol.

[17] Murakami N, Kasamatsu T, Wakita A, Nakamura S, Okamoto H, Inaba K (2014). CT based three dimensional dose–volume evaluations for high-dose rate intracavitary brachytherapy for cervical cancer. BMC Cancer.

[18] Ribeiro I, Janssen H, De Brabandere M, Nulens A, De Bal D, Vergote I (2016). Long term experience with 3D image guided brachytherapy and clinical outcome in cervical cancer patients. Radiother Oncol.

[19] Sturdza A, Pötter R, Fokdal LU, Haie-Meder C, Tan LT, Mazeron R (2016). Image guided brachytherapy in locally advanced cervical cancer: improved pelvic control and survival in RetroEMBRACE, a multicenter cohort study. Radiother Oncol.

[20] Mazeron R, Fokdal LU, Kirchheiner K, Georg P, Jastaniyah N, Šegedin B (2016). Dose-volume effect relationships for late rectal morbidity in patients treated with chemoradiation and MRI-guided adaptive brachytherapy for locally advanced cervical cancer: results from the prospective multicenter EMBRACE study. Radiother Oncol.

[21] Kim Y, Kim YJ, Kim JY, Lim YK, Jeong C, Jeong J (2017). Toxicities and dose-volume histogram parameters of MRI-based brachytherapy for cervical cancer. Brachytherapy.

[22] Pötter R, Tanderup K, Kirisits C, de Leeuw A, Kirchheiner K, Nout R, et al. The EMBRACE II study: The outcome and prospect of two decades of evolution within the GEC-ESTRO GYN working group and the EMBRACE studies. Clin Transl Radiat Oncol. 2018;9:48–60. 10.1016/j.ctro.2018.01.001 PMC5862686 29594251

[23] Scholz M, Kraft G (1996). Track structure and the calculation of biological effects of heavy charged particles. Adv Space Res.

[24] Oike T, Niimi A, Okonogi N, Murata K, Matsumura A, Noda SE (2016). Visualization of complex DNA double-strand breaks in a tumor treated with carbon ion radiotherapy. Sci Rep.

[25] Grün R, Friedrich T, Elsässer T, Krämer M, Zink K, Karger CP (2012). Impact of enhancements in the local effect model (LEM) on the predicted RBE-weighted target dose distribution in carbon ion therapy. Phys Med Biol.

