# Supplementary material for: Dose constraints in the rectum and bladder following carbon-ion radiotherapy for uterus carcinoma: a retrospective pooled analysis
Source: Radiat Oncol. 2018 Jun 25;13:119. doi: 10.1186/s13014-018-1061-7 (PMC6019512; doi:10.1186/s13014-018-1061-7)
Supplement: Supplementary file 1 — Table S1. Dose fractions of C-ion RT and the number of patients with morbidities for each prescription dose. The number in brackets indicates the number of patients who were treated with a previous dose constraint of < 60 Gy (RBE), maximal dose, to the GI tract. For those patients with both rectum and bladder morbidities, the patient was categorized as having a higher grade cancer in this table. (DOCX 32 kb) [file 13014_2018_1061_MOESM1_ESM.docx]

Table 1S. Dose fractions of C-ion RT and the number of patients with morbidities for each prescription dose.

| **Dose fractions** | **Total**  **n = 132** | RTOG/EORTC Grade | | | | |
| --- | --- | --- | --- | --- | --- | --- |
|  |  | **0** | **1** | **2** | **3** | **4** |
| **24 fractions**  52.8 Gy (RBE) /24 fractions/6 weeks  57.6 Gy (RBE) /24 fractions/6 weeks  62.4 Gy (RBE) /24 fractions/6 weeks  67.2 Gy (RBE) /24 fractions/6 weeks  68.8 Gy (RBE) /24 fractions/6 weeks  72.0 Gy (RBE) /24 fractions/6 weeks  　72.8 Gy (RBE) /24 fractions/6 weeks  **20 fractions**  62.4 Gy (RBE) /20 fractions/5 weeks  64.0 Gy (RBE) /20 fractions/5 weeks  64.8 Gy (RBE) /20 fractions/5 weeks  68.0 Gy (RBE) /20 fractions/5 weeks  68.8 Gy (RBE) /20 fractions/5 weeks  71.2 Gy (RBE) /20 fractions/5 weeks  72.0 Gy (RBE) /20 fractions/5 weeks  74.4 Gy (RBE) /20 fractions/5 weeks | 6  5  5  5  6  7  7  6  11  5  17  1  26  6  19 | 3  4  1  0  4  5  1  5 (0)  5 (0)  2 (0)  11 (11)  0 (0)  22 (22)  5 (5)  10 (10) | 3  1  2  2  2  0  2  1 (0)  3 (0)  3 (1)  3 (3)  0 (0)  0 (0)  1 (1)  8 (8) | 0  0  1  0  0  0  2  0 (0)  3 (0)  0 (0)  2 (2)  1 (0)  4 (4)  0 (0)  1 (1) | 0  0  0  0  0  0  1  0 (0)  0 (0)  0 (0)  0 (0)  0 (0)  0 (0)  0 (0)  0 (0) | 0  0  1  3  0  2  1  0 (0)  0 (0)  0 (0)  1 (1)  0 (0)  0 (0)  0 (0)  0 (0) |

Abbreviation:

RTOG/EORTC: Radiation Therapy Oncology Group/European Organization for Research and Treatment of Cancer.
