# Supplementary figures and images for: Dose constraints in the rectum and bladder following carbon-ion radiotherapy for uterus carcinoma: a retrospective pooled analysis
Source: Radiat Oncol. 2018 Jun 25;13:119. doi: 10.1186/s13014-018-1061-7 (PMC6019512; doi:10.1186/s13014-018-1061-7)

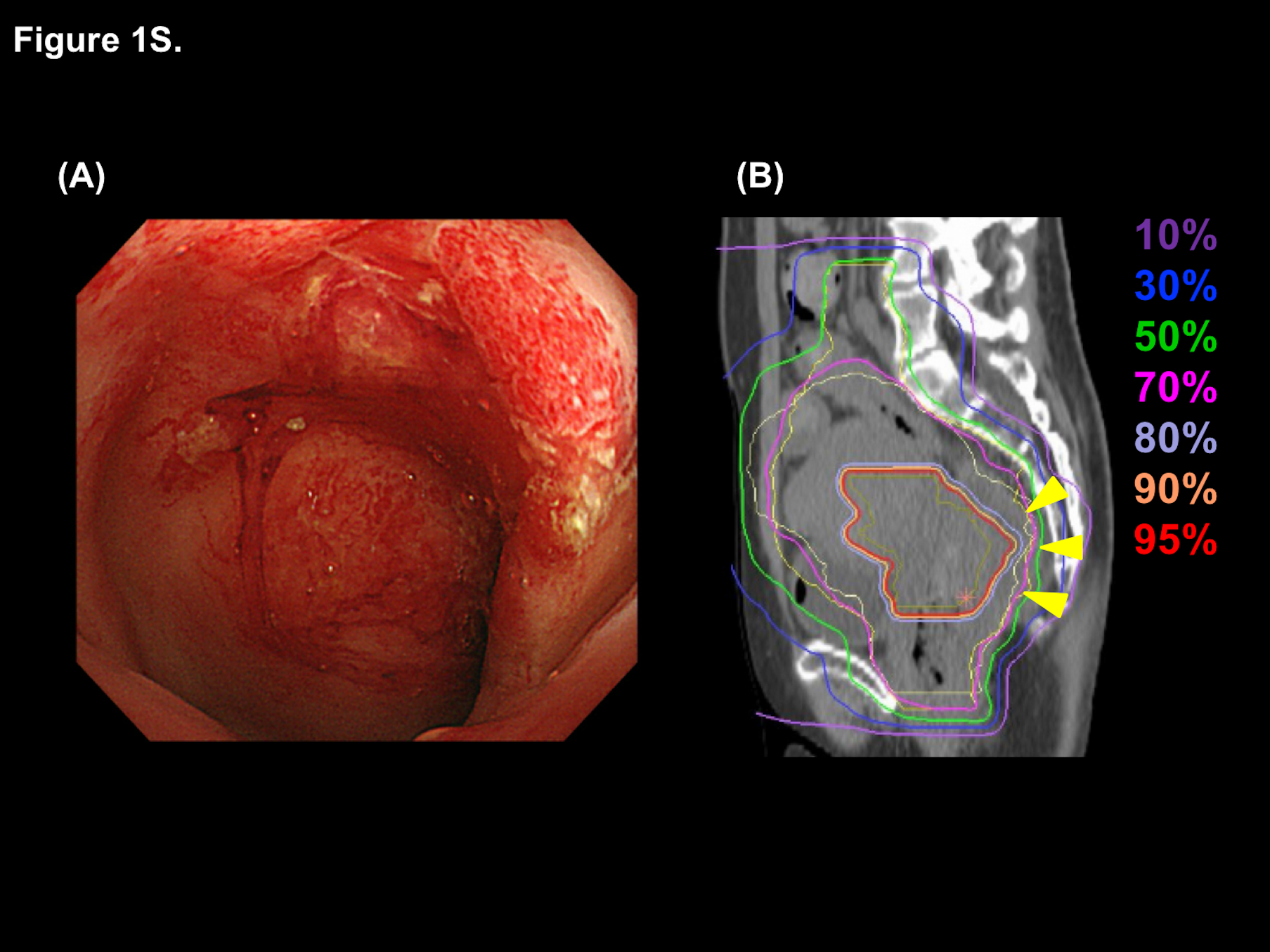

Supplement: Supplementary file 2 — Figure S1. Representative endoscopic image of proctitis and isodose curves of carbon-ion radiotherapy of the patient. (A) Representative endoscopic image of grade 2 proctitis and (B) isodose curves of carbon-ion radiotherapy on a sagittal computed tomography image of the patient. This patient developed Grade 2 proctitis on the anterior rectal wall 1 year after carbon-ion radiotherapy. Yellow arrows indicate the proctitis region. Highlighted are 95% (red), 90% (orange), 80% (light purple), 70% (pink), 50% (green), 30% (green), and 10% (purple) isodose curves. (TIFF 9568 kb) [file 13014_2018_1061_MOESM2_ESM.tiff]

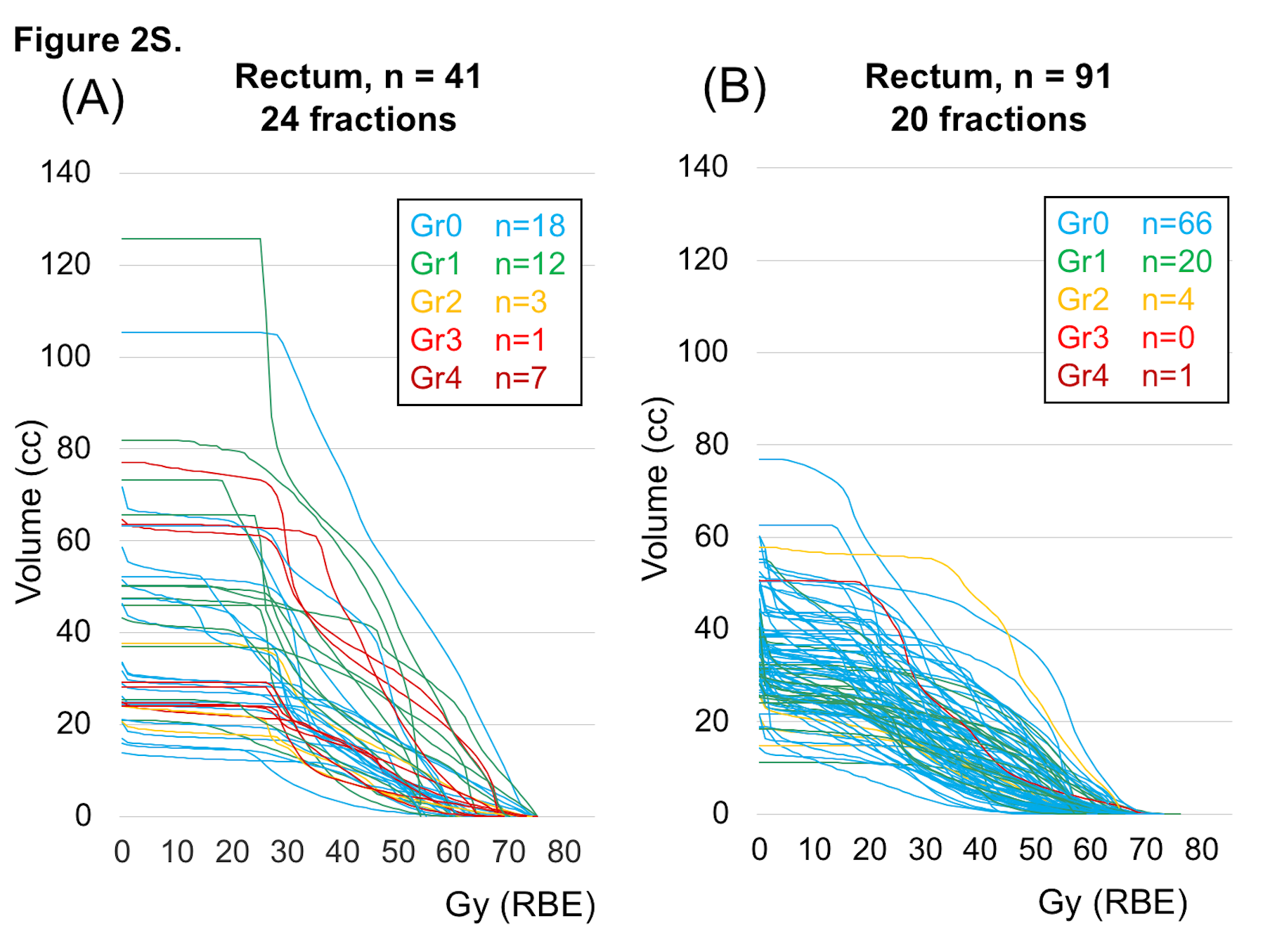

Supplement: Supplementary file 3 — Figure S2. Dose–volume histograms of the rectum. (A) Dose–volume histograms (DVHs) of the rectum in 41 patients who received carbon-ion radiotherapy in 24 fractions and (B) DVHs of the rectum in 91 patients who received carbon-ion radiotherapy in 20 fractions. Each line shows the data for each patient and each color indicates the severity of proctitis; Grade 0 (cyan), Grade 1 (green), Grade 2 (yellow), Grade 3 (red), and Grade 4 (brown). Abbreviation: Gr = Grade, RBE = relative biological effectiveness. (TIFF 9568 kb) [file 13014_2018_1061_MOESM3_ESM.tiff]

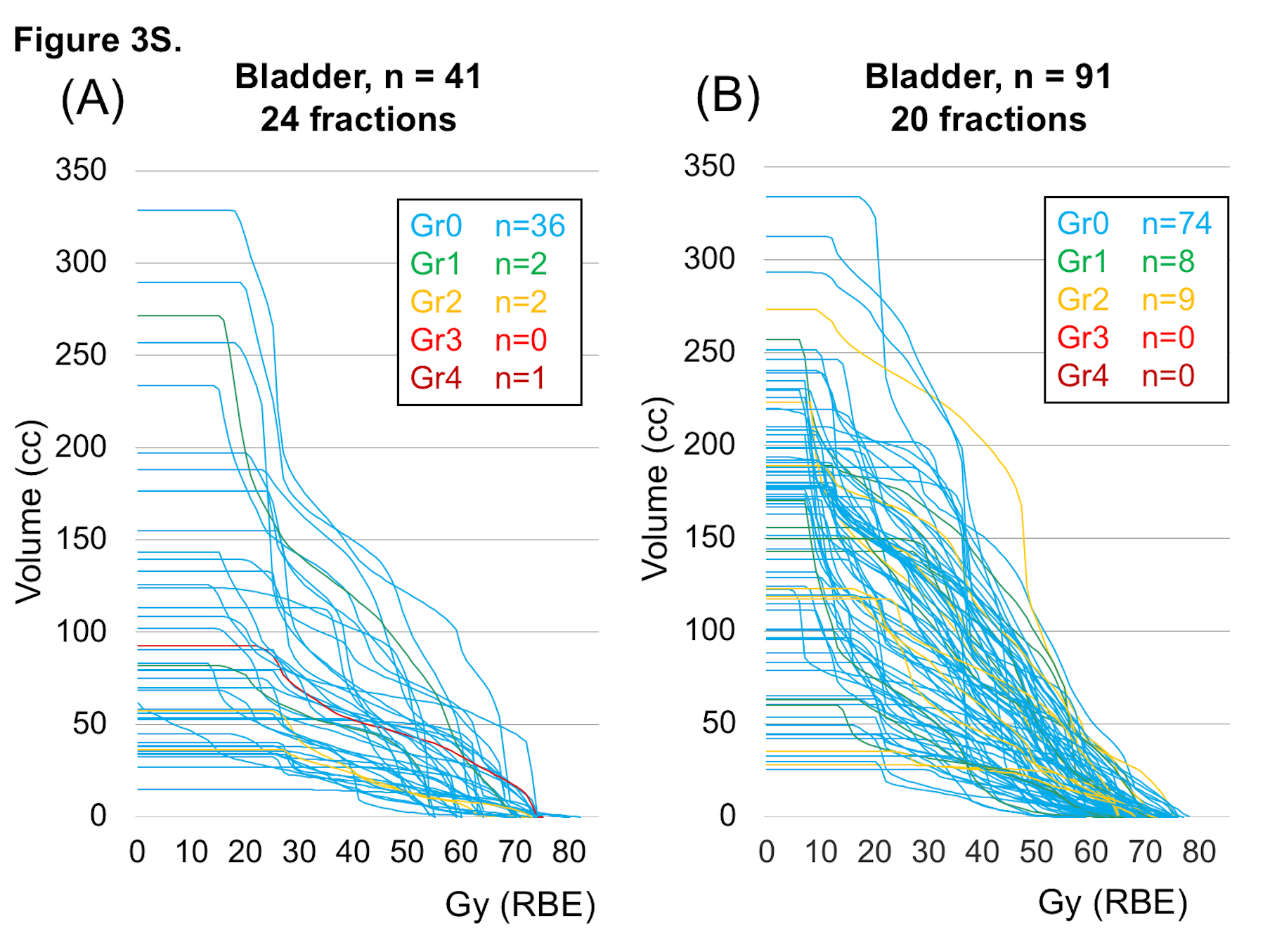

Supplement: Supplementary file 4 — Figure S3. Dose–volume histograms of the bladder. (A) Dose–volume histograms (DVHs) of the bladder in 41 patients who received carbon-ion radiotherapy in 24 fractions and (B) DVHs of the bladder in 91 patients who received carbon-ion radiotherapy in 20 fractions. Each line shows the data for each patient and each color indicates the severity of cystitis; Grade 0 (cyan), Grade 1 (green), Grade 2 (yellow), Grade 3 (red), and Grade 4 (brown). Abbreviation: Gr = Grade, RBE = relative biological effectiveness. (TIFF 9568 kb) [file 13014_2018_1061_MOESM4_ESM.tiff]

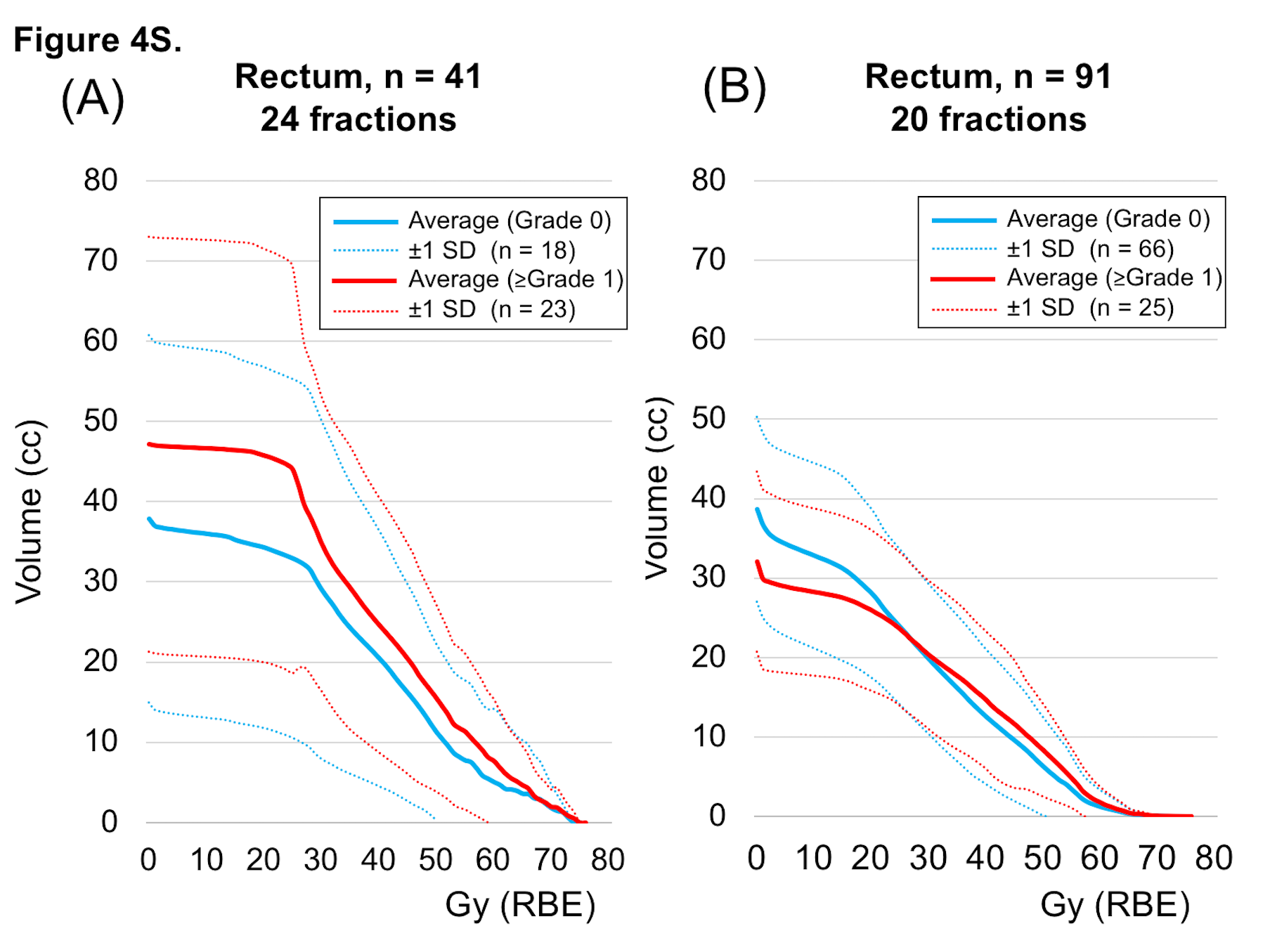

Supplement: Supplementary file 6 — Figure S4. Comparisons of dose–volume histograms of the rectum. Comparisons of dose–volume histograms of the rectum (A) in 41 patients who received carbon-ion radiotherapy in 24 fractions and (B) in 91 patients who received carbon-ion radiotherapy in 20 fractions. Each line shows the average value and each color indicates the presence or absence of proctitis; Grade 0 (cyan) and ≥ Grade 1 (red). Solid lines show the averages and dotted lines show the ±1 SD. Abbreviation: SD = standard deviation. (TIFF 9568 kb) [file 13014_2018_1061_MOESM6_ESM.tiff]

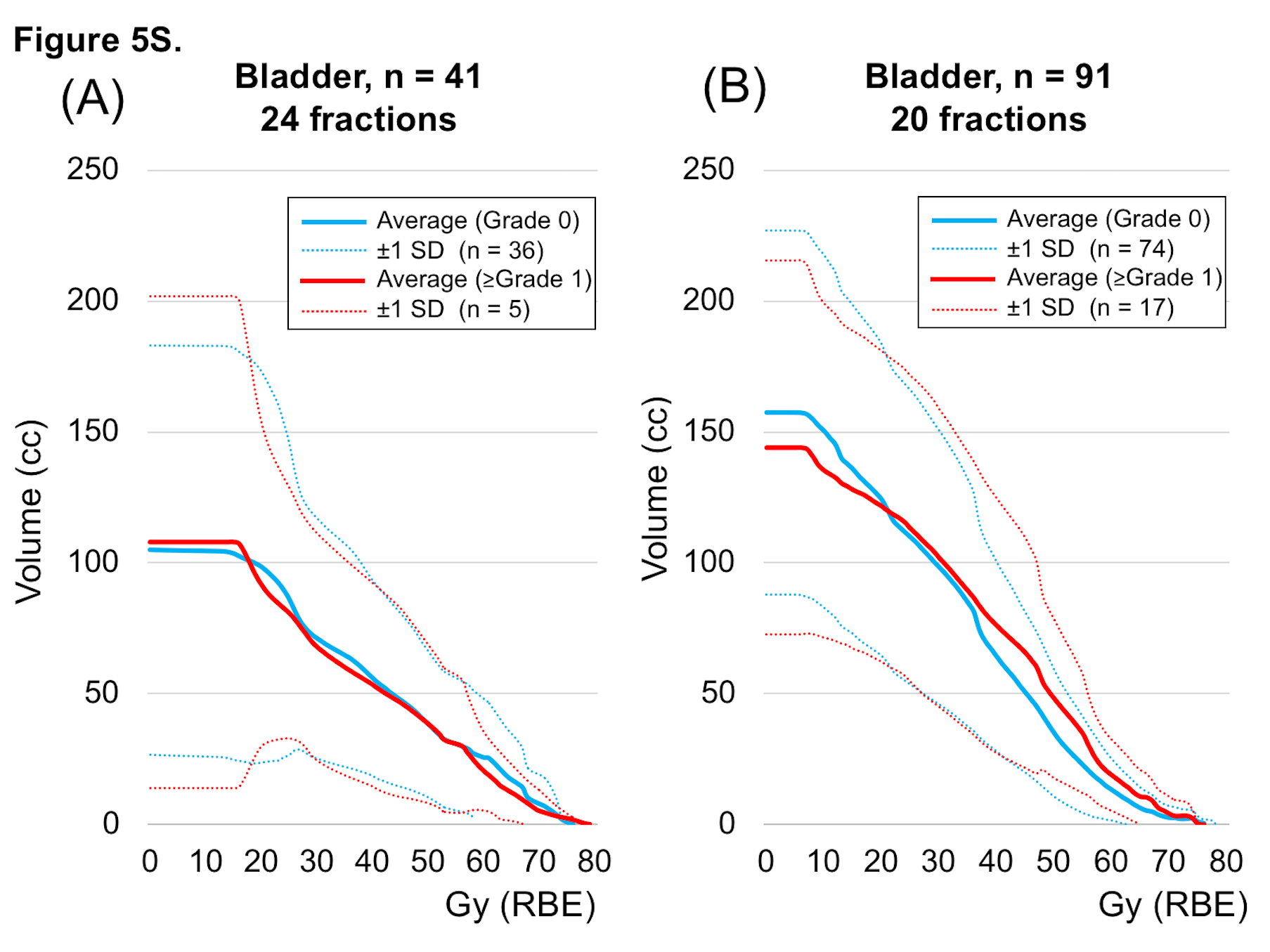

Supplement: Supplementary file 7 — Figure S5. Comparisons of dose–volume histograms of the bladder. Comparisons of dose–volume histograms of the bladder (A) in 41 patients who received carbon-ion radiotherapy in 24 fractions and (B) in 91 patients who received carbon-ion radiotherapy in 20 fractions. Each line shows the average value and each color indicates the presence or absence of cystitis; Grade 0 (cyan) and ≥ Grade 1 (red). Solid lines show the averages and dotted lines show the ±1 SD. Abbreviation: SD = standard deviation. (TIFF 9568 kb) [file 13014_2018_1061_MOESM7_ESM.tiff]
