# Supplementary material for: Dose constraints in the rectum and bladder following carbon-ion radiotherapy for uterus carcinoma: a retrospective pooled analysis
Source: Radiat Oncol. 2018 Jun 25;13:119. doi: 10.1186/s13014-018-1061-7 (PMC6019512; doi:10.1186/s13014-018-1061-7)
Supplement: Supplementary file 5 — Table S2. List of patients who developed grade 2 or higher morbidities. Abbreviation: UCC: Uterine cervical carcinoma, UEA: Uterine endometrioid adenocarcinoma. The asterisks indicates the number of patients who were treated with a previous dose constraint of < 60 Gy (RBE), maximal dose, to the GI tract. (DOCX 19 kb) [file 13014_2018_1061_MOESM5_ESM.docx]

Table 2S. 　List of patients who developed grade 2 or higher morbidities.

| **Primary disease** | **Stage** | **Age** | **RT dose** | **D_2cc_ rectum** | **D_5cc_ bladder** | **Site, Grade** | **Onset** | **Detail** |
| --- | --- | --- | --- | --- | --- | --- | --- | --- |
|  |  | **(years)** | **Gy (RBE)** | **Gy (RBE)** | **Gy (RBE)** |  | **(months)** |  |
| **24 fractions** | |  |  |  |  |  |  |  |
| UCC | T3bN1M0 | 56 | 62.4 | 54.3 | 68.8 | Rectum, 2 | 10 | Bleeding |
| UCC | T4N0M0 | 51 | 62.4 | 63.3 | 63.7 | Rectum, 4 | 11 | Recto-vaginal fistula |
| UCC | T4N0M0 | 55 | 67.2 | 68.2 | 68.2 | Rectum, 4 | 17 | Recto-vaginal fistula |
| UCC | T3bN0M0 | 68 | 67.2 | 66.3 | 69.1 | Rectum, 4 | 26 | Perforation |
| UCC | T4N0M0 | 64 | 67.2 | 67.7 | 68.0 | Rectum, 4 | 32 | Perforation |
| UCC | T3bN1M0 | 50 | 72 | 63.7 | 56.7 | Rectum, 4 | 12 | Perforation |
| UCC | T3bN1M0 | 48 | 72 | 63.8 | 68.3 | Rectum, 4 | 13 | Recto-vaginal fistula |
|  |  |  |  |  |  | Bladder, 2 | 22 | Hematuria |
| UCC | T3bN1M0 | 77 | 72.8 | 67.5 | 72.6 | Rectum, 2 | 9 | Bleeding |
| UCC | T3bN0M0 | 76 | 72.8 | 59.6 | 67.6 | Rectum, 2 | 10 | Bleeding |
| UCC | T3bN1M0 | 48 | 72.8 | 65.5 | 61.2 | Rectum, 3 | 30 | Massive Bleeding |
|  |  |  |  |  |  | Bladder, 2 | 37 | Hematuria |
| UCC | T4N0M0 | 64 | 72.8 | 58.5 | 73.1 | Rectum, 4 | 12 | Perforation |
|  |  |  |  |  |  | Bladder, 4 | 12 | Vesicovaginal fistula |
| **20 fractions** | |  |  |  |  |  |  |  |
| UCC | T2bN1M0 | 75 | 64 | 64.7 | 55.4 | Rectum, 2 | 11 | Bleeding |
| UCC | T4N0M0 | 52 | 64 | 57.8 | 64.5 | Bladder, 2 | 25 | Hematuria |
| UCC | T3bN1M0 | 57 | 64 | 50.4 | 64.1 | Bladder, 2 | 17 | Hematuria |
| UCC* | T3bN1M0 | 54 | 68 | 55.6 | 65.2 | Bladder, 2 | 19 | Hematuria |
| UCC* | T3bN0M0 | 76 | 68 | 50.1 | 65.1 | Bladder, 2 | 98 | Bladder atrophy |
| UCC* | T2bN0M0 | 37 | 68 | 64.0 | 67.6 | Rectum, 4 | 14 | Recto-vaginal fistula |
| UCC | T3bN1M0 | 52 | 68.8 | 59.1 | 68.2 | Bladder, 2 | 25 | Hematuria |
| UCC* | T3bN0M0 | 76 | 71.2 | 55.1 | 70.2 | Bladder, 2 | 9 | Hematuria |
| UCC* | T3bN1M0 | 49 | 71.2 | 53.5 | 65.1 | Bladder, 2 | 20 | Hematuria |
| UEA* | T2bN0M0 | 73 | 71.2 | 57.0 | 62.3 | Rectum, 2 | 6 | Bleeding |
|  |  |  |  |  |  | Bladder, 2 | 12 | Hematuria |
| UEA* | T2bN0M0 | 64 | 71.2 | 53.6 | 49.2 | Rectum, 2 | 10 | Bleeding |
| UCC* | T3bN1M0 | 74 | 74.4 | 51.9 | 74.3 | Rectum, 2 | 18 | Bleeding |
|  |  |  |  |  |  | Bladder, 2 | 18 | Hematuria |

Abbreviation:

UCC: Uterine cervical carcinoma, UEA: Uterine endometrioid adenocarcinoma
