# Supplementary material for: Dose constraints in the rectum and bladder following carbon-ion radiotherapy for uterus carcinoma: a retrospective pooled analysis
Source: Radiat Oncol. 2018 Jun 25;13:119. doi: 10.1186/s13014-018-1061-7 (PMC6019512; doi:10.1186/s13014-018-1061-7)
Supplement: Supplementary file 8 — Table S3. Correlation between dose-volume histogram parameters and late morbidities of the rectum in the 24 fractions group. This table summarizes the dose–volume histogram parameters from V10 to V60, D5cc, D2cc, and Dmax according to the grading of rectal morbidity in the 24 fractions group. (DOCX 16 kb) [file 13014_2018_1061_MOESM8_ESM.docx]

Table 3S. Correlation between dose-volume histogram parameters and late morbidities in rectum in 24 fractions group.

|  | **24 fractions** | | | | | |
| --- | --- | --- | --- | --- | --- | --- |
|  | **Grade 0-1** | **≥Grade 2** | ***p* value** | **≤Grade 3** | **Grade 4** | ***p* value** |
| Rectum | n = 30 | n = 11 |  | n = 34 | n = 7 |  |
| V10 (mean ± SD, cc)  V20 (mean ± SD, cc)  V30 (mean ± SD, cc)  V40 (mean ± SD, cc)  V50 (mean ± SD, cc)  V60 (mean ± SD, cc)  D5 cc [mean ± SD, Gy (RBE)]  D2 cc [mean ± SD, Gy (RBE)]  Dmax [mean ± SD, Gy (RBE)] | 43.8 ± 26.6  42.2 ± 26.5  33.1 ± 20.5  23.9 ± 16.9  14.2 ± 12.1  7.2 ± 9.2  56.0 ± 8.3  59.8 ± 7.4  64.9 ± 7.6 | 37.2 ± 20.1  36.7 ± 19.9  30.2 ± 17.6  20.1 ± 13.2  12.4 ± 9.8  5.9 ± 6.4  56.4 ± 7.8  63.5 ± 4.4  69.8 ± 3.8 | 0.470  0.545  0.687  0.515  0.668  0.676  0.893  0.136  0.053 | 41.7 ± 25.8  40.2 ± 25.6  31.6 ± 19.8  22.6 ± 16.3  13.4 ± 11.6  6.5 ± 8.6  55.7 ± 8.1  60.1 ± 7.2  65.6 ± 7.2 | 43.7 ± 22.5  43.1 ± 22.1  36.0 ± 19.5  24.1 ± 15.0  15.2 ± 11.2  7.5 ± 7.5  58.1 ± 8.2  64.5 ± 3.3  68.9 ± 2.3 | 0.853  0.787  0.604  0.828  0.716  0.782  0.496  0.132  0.249 |
